# Novel histopathologic predictors for renal outcomes in crescentic glomerulonephritis

**DOI:** 10.1371/journal.pone.0236051

**Published:** 2020-07-27

**Authors:** Jeong-Hoon Lim, Man-Hoon Han, Yong-Jin Kim, Yena Jeon, Hee-Yeon Jung, Ji-Young Choi, Jang-Hee Cho, Chan-Duck Kim, Yong-Lim Kim, Hajeong Lee, Dong Ki Kim, Kyung Chul Moon, Sun-Hee Park

**Affiliations:** 1 Department of Internal Medicine, School of Medicine, Kyungpook National University, Kyungpook National University Hospital, Daegu, South Korea; 2 Department of Pathology, School of Medicine, Kyungpook National University, Kyungpook National University Hospital, Daegu, South Korea; 3 Department of Statistics, Kyungpook National University, Daegu, South Korea; 4 Department of Internal Medicine, Seoul National University College of Medicine, Seoul, South Korea; 5 Department of Pathology, Seoul National University College of Medicine, Seoul, South Korea; International University of Health and Welfare, School of Medicine, JAPAN

## Abstract

**Introduction:**

Crescentic glomerulonephritis (CrGN) is a histologic feature of severe glomerular injury, clinically characterized by a rapid decline of renal function when not treated in a timely fashion. Factors associated with CrGN prognosis have not been thoroughly investigated. This study investigated the prognostic predictors of renal outcomes associated with CrGN, such as the histopathologic classification of anti-neutrophil cytoplasmic antibody (ANCA)-associated glomerulonephritis, arteriosclerosis, and tertiary lymphoid organ (TLO) formation.

**Methods:**

A total of 114 patients diagnosed with CrGN between 2010 and 2018 at two university-based hospitals has been retrospectively analyzed. Relationships between potential predictors and renal outcomes were analyzed using Cox proportional hazards model and linear regression analysis.

**Results:**

The mean age was 61.0 ± 15.3 years, and 49.1% were male. Among them, 92 (80.7%) and 11 (9.6%) patients were positive for ANCA and for anti-glomerular basement membrane antibody, respectively. During the median follow-up of 458.0 days, 55 patients (48.2%) had advanced to end-stage renal disease (ESRD). Cox proportional hazards analysis revealed that patients under the mixed and sclerotic classes had worse renal survival compared to those in the focal class (mixed: hazard ratio [HR], 3.74; 95% confidence interval [CI], 1.18 to 11.82; *P* = 0.025; sclerotic: HR, 4.84; 95% CI, 1.44 to 16.32; *P* = 0.011). Severe arteriosclerosis was also associated with poor renal survival (HR, 2.44; 95% CI, 1.04 to 5.77; *P* = 0.042). TLOs were observed in 41 patients (36.0%). Moreover, TLO formation was also a prognostic factor for ESRD (HR, 1.82; 95% CI, 1.03 to 3.21; *P* = 0.040). In the multivariate linear regression analysis, age and sclerotic class were independent predictors for the change in estimated glomerular filtration rate during 1 year after biopsy.

**Conclusions:**

Specific histopathologic findings, histopathologic classification, severity of arteriosclerosis, and TLO formation provide helpful information in predicting renal outcomes associated with CrGN.

## Introduction

Crescentic glomerulonephritis (CrGN), characterized by the presence of glomerular crescents observed using histologic examination, is a pattern of injury associated with a high risk of renal failure [1, 2]. CrGN is classified into three types according to their pathogenic mechanisms: anti-glomerular basement membrane (GBM) disease, immune complex-mediated disease, and pauci-immune disease. Although the mechanisms initiating inflammation and renal parenchymal destruction vary according to disease type, advanced glomerular changes and renal outcomes are similar among the types [3, 4]. Therefore, the classes and proportions of crescentic glomeruli are considered more important than the CrGN types in predicting CrGN outcomes and management [5, 6].

Prompt diagnosis and earlier initiation of immunosuppressive therapy are essential in improving renal survival, but the risks associated with immunosuppressive therapy must be balanced against benefits [3, 7, 8]. Therefore, it is important to predict prognosis and reversibility at the time of diagnosis to determine the appropriate timing and extent of immunosuppression. Previous studies showed that various factors are associated with renal outcomes in CrGN, which include age, renal function at baseline, histopathologic classification, and degree of tubular atrophy [9, 10]; however, most of these factors have not yet been validated. The histopathologic classification of anti-neutrophil cytoplasmic antibody (ANCA)-associated glomerulonephritis (AAGN), divided into four classes—focal, crescentic, mixed, and sclerotic—has provided prognostic values for renal outcomes, and it has been validated by a number of subsequent studies [9, 11, 12]. Moreover, the histopathologic classification has recently been identified as a predictor for renal outcomes among CrGN patients in Europe [4]; however, it has not yet been validated by other studies.

Since other known risk factors are insufficient in predicting the prognosis of CrGN patients, this study focused on chronic inflammation and histopathologic vascular changes. Lymphoid neogenesis, known as tertiary lymphoid organ (TLO) formation, indicates nodular inflammatory cell infiltration in non-lymphoid organs [13–15]. TLOs are characterized histopathologically by aggregated small cells, such as B and T lymphocytes, and other cellular components consisting of larger follicular structures [16, 17]. TLOs have been identified in chronic inflammatory conditions involving various organs, such as Grave’s disease (thyroid gland), rheumatoid arthritis (joints), multiple sclerosis (central nervous system), glomerulonephritis (kidney), and chronic rejection in organ transplantation (heart, lung, and kidney) [16, 18]; these are also associated with various inflammatory renal diseases, such as IgA nephropathy, membranous nephropathy, graft rejection, and lupus nephritis [17–19]. TLO formation is indicative of a chronic inflammatory status and promote local immune responses; therefore, TLOs play important roles in disease progression [17]. Additionally, chronic vascular changes, such as atherosclerosis, indicate underlying chronic conditions, such as aging, hypertension, diabetes, obesity, hyperlipidemia, and chronic inflammation. Such changes lead to ischemic parenchymal atrophy, tubular damage, interstitial fibrosis, and glomerular injury [20, 21].

This study aimed to validate the histopathologic classification in predicting renal outcomes and evaluate whether the TLO formation and severity of arteriosclerosis could further help predict renal outcomes among CrGN patients.

## Materials and methods

### Patients

A total of 114 CrGN patients from two university-based hospitals between March 2004 and March 2018 were enrolled in this study. Patients whose biopsy specimens had less than 10 glomeruli and those who were lost to follow-up within 1 year were excluded in this study. The study protocol was reviewed and approved by the institutional review boards of Kyungpook National University Hospital (2017-08-013-003) and Seoul National University Hospital (H1802-102-924). Informed consent was waived because there was no infringement on the privacy or health of the patients which occurred during the study. Patient data were anonymized and de-identified prior to the analyses.

### Data collection

Patient demographic data, comorbid diseases, and laboratory data were collected at the time of renal biopsy. Repeat serum creatinine was measured 1 year after the biopsy was taken. Estimated glomerular filtration rate (eGFR) was calculated using the CKD-EPI equation [22]. Renal replacement therapy initiation date, deaths, and last follow-up data were also captured.

**Fig 1:**
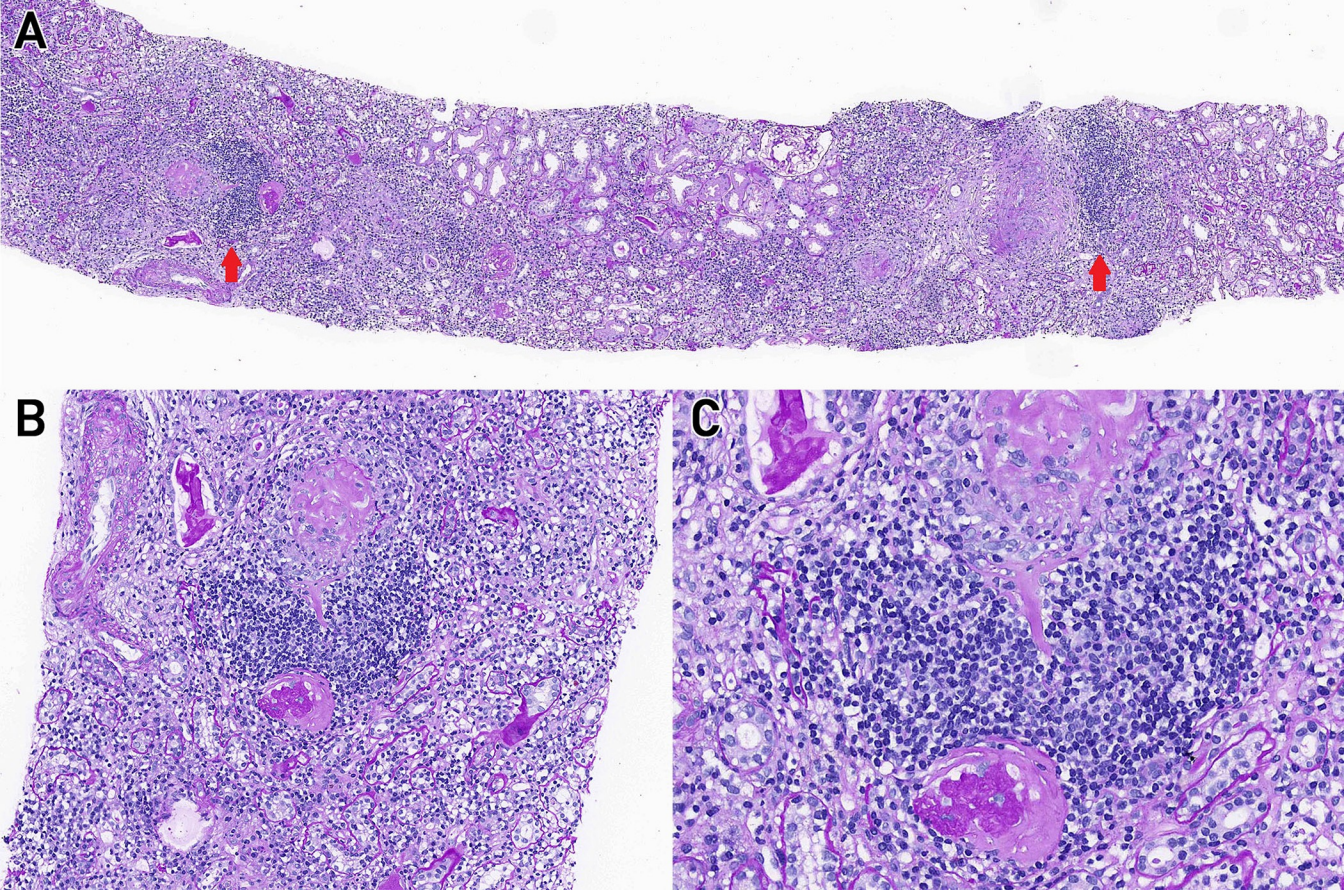
Tertiary lymphoid organ formation in renal parenchyma. PAS staining reveals nodular lymphoid aggregates. (A) Arrows indicate tertiary lymphoid organ formation. Original magnification ×50. (B) Original magnification ×200. (C) Original magnification ×400. Abbreviation: PAS, periodic acid-Schiff.

All renal biopsy specimens were independently reanalyzed by two renal pathologists who were blinded to the patient information. Analyzed histopathologic details include the following: number of total glomeruli (and separate tallies for normal, crescentic [cellular and fibrous], and globally sclerotic glomeruli), histopathologic classification using the algorithm developed by Berden et al. to categorize AAGN [9], severity of arteriosclerosis, TLO formation (Fig 1), severity of tubular atrophy, peritubular capillaritis, tubulitis, and interstitial inflammation [23, 24]. The histopathologic classification has identified four classes: focal, crescentic, mixed, and sclerotic. Specimens consisting of more than 50% normal glomeruli were classified as focal, those with more than 50% cellular crescentic glomeruli were classified as crescentic, and those with more than 50% of their glomeruli having a globally sclerotic appearance were classified as sclerotic (S1 Fig). If the specimens could not be neatly categorized using the aforementioned classes, they were classified as mixed. The kappa value (κ = 0.90) reflecting the interobserver agreement with the histopathologic classification indicated excellent concordance. The discrepancies between the two pathologists were resolved through consensus meetings according to the recommendation [25]. Renal arteriosclerosis was evaluated using a semiquantitative scoring method based on the extent of occlusion of the most severely affected vessel [23]. Scores of 0, 1, 2, and 3 reflected arterial luminal narrowing of less than 10%, 10% to 25%, 26% to 50%, and more than 50%, respectively (S2 Fig). TLO formation was defined as an organized cluster composed of lymphocytes with an arbitrary cut-off of 50 cells [26]. Specimens that exhibited tubular atrophy in more than 50% of the cortical tubules were defined as having severe tubular atrophy [10, 23]. Immunofluorescence findings for IgG, IgA, IgM, C3, and fibrinogen were graded on an intensity scale from 0 to 4+.

### Outcomes

Renal survival was defined as the time from renal biopsy to the start of the renal replacement therapy (more than 3 months). Renal replacement therapy was defined as hemodialysis, peritoneal dialysis, or kidney transplantation. Patient survival was defined as the time from renal biopsy to death from any cause.

### Treatment

The individual treatment information for CrGN was not available in our data. Based on the clinician’s experience, patient’s condition, and previously reported treatment protocols [27, 28], clinicians established treatment plans at the time of diagnosis. In general, patients received high-dose intravenous methylprednisolone (500 mg/day) for 3 days, and pulse intravenous or daily oral cyclophosphamide was used in combination for induction. After receiving high-dose corticosteroids, patients were prescribed oral prednisolone 1 mg/kg/day (not exceeding 60 mg daily) for 4–6 weeks, with tapering to 5–10 mg/day within 3–4 months. Low-dose corticosteroids and oral azathioprine were used for maintenance therapy. Patients with other organ- or life-threatening involvement, such as diffuse alveolar hemorrhage or serum creatinine >500 μmol/L (5.7 mg/dL) due to rapidly progressive glomerulonephritis, additionally received plasma exchanges as induction. Rituximab was administered to 24 patients (21.1%) as induction therapy since 2011 after two studies have demonstrated its efficacy [29, 30].

### Statistical analysis

The Kolmogorov–Smirnov test was used to evaluate if variables were normally distributed. Continuous variables are presented as mean ± standard deviation or median (interquartile range [IQR]) according to their distribution, and categorical variables are presented as number and percentage (%). Student’s *t*-tests were used to compare continuous variables, and Pearson chi-square tests or Fisher’s exact tests were used to compare categorical variables as appropriate. The kappa statistic was used to measure differences in histopathologic classification between two renal pathologists. Renal survival and patient survival were analyzed using Kaplan–Meier analysis, and log-rank tests were used to analyze the differences across categories of variables. Multivariate Cox proportional hazards regression analyses were performed for the variables potentially associated with renal and patient survival. Multivariable linear regression was used to evaluate the association between potential predictors of follow-up renal function and changes of eGFR during 1 year after biopsy. Potential predictors were designated according to the findings of previous studies: age, sex, comorbid diabetes and hypertension, eGFR at biopsy, histopathologic classification, severity of arteriosclerosis, TLO formation, and severe tubular atrophy [10, 18, 31, 32]. Univariate logistic regression analyses were performed to identify associated factors with TLO formation. The statistical analyses were performed using SPSS Statistics for Windows, Version 22 (IBM Corp., Armonk, NY, USA). A *P* value less than 0.05 was considered statistically significant.

## Results

### Baseline clinical and histopathologic characteristics

Among the 114 patients, the mean age was 61.0 ± 15.3 years, and 49.1% were males (Table 1). Ninety-two patients (80.7%) were positive for ANCA, and most of them were myeloperoxidase-ANCA-positive (75.4%). Eleven patients (9.6%) were positive for anti-GBM antibody and four patients (3.5%) were positive both for ANCA and anti-GBM antibody; nine patients (7.9%) had immune complex-mediated glomerulonephritis, such as membranoproliferative glomerulonephritis (3 patients), IgA nephropathy (5 patients), and lupus nephritis (1 patient) (S1 Table). During the median follow-up of 458.0 (IQR, 92.5–1254.5) days, 55 patients (48.2%) reached end-stage renal disease (ESRD). The positive ratios for ANCA and anti-GBM antibody were similar between ESRD and non-ESRD patients. Patients who progressed to ESRD were older than non-ESRD patients (*P* = 0.037) and were more likely to have comorbid hypertension at the time of diagnosis (*P* = 0.026). Mean baseline blood urea nitrogen and serum creatinine levels were higher and eGFR was lower among ESRD patients (all *P* < 0.001), but there was no between-group difference in terms of C-reactive protein level.

**Table 1:** Baseline clinical and histopathologic characteristics.

Variables	Total (n = 114)	ESRD (n = 55)	Non-ESRD (n = 59)	*P* value
Age (years)	61.0 ± 15.3	64.0 ± 14.5	58.0 ± 15.7	**0.037**
Sex (% male)	56 (49.1)	29 (52.7)	27 (45.8)	0.457
Comorbid disease, n (%)				
Diabetes	14 (12.3)	6 (10.9)	8 (13.6)	0.667
Hypertension	44 (38.6)	27 (49.1)	17 (28.8)	**0.026**
Follow-up duration after biopsy (days)	458.0 (92.5–1254.5)	386.0 (77.0–1317.0)	681.5 (134.3–1332.5)	0.287
Laboratory data				
Blood urea nitrogen (mg/dL)	46.0 (31.0–65.6)	57.2 (42.4–79.0)	37.0 (27.5–58.0)	**<0.001**
Creatinine (mg/dL)	4.1 (2.2–5.9)	5.2 (4.0–7.8)	2.6 (1.6–4.3)	**<0.001**
Estimated GFR (mL/min/1.73 m^2^)	13.1 (8.2–25.7)	8.6 (6.7–13.2)	21.5 (12.9–36.1)	**<0.001**
CRP (mg/dL)	2.8 (0.5–9.2)	2.0 (0.3–6.3)	3.4 (0.8–9.8)	0.150
Spot urine protein-to-creatinine ratio (g/g)	2.4 (1.5–4.2)	2.5 (1.6–4.9)	2.3 (1.5–3.9)	0.319
Immunology, n (%)				
ANCA	92 (80.7)	45 (81.8)	47 (79.7)	0.771
MPO-ANCA	86 (93.5)	41 (91.1)	45 (95.7)	0.831
PR3-ANCA	6 (6.5)	4 (8.9)	2 (4.3)	0.427
Anti-GBM antibody	11 (9.6)	5 (9.1)	6 (10.2)	0.845
Histopathologic findings				
Number of glomeruli	16.0 (12.0–22.3)	16.0 (12.0–24.0)	16.0 (12.0–22.0)	0.337
Normal glomeruli (%)	21.6 (8.9–40.0)	9.1 (0.0–23.1)	33.3 (20.0–50.0)	**<0.001**
Crescentic glomeruli (%)	51.3 (30.8–70.6)	56.0 (33.3–76.0)	47.8 (30.0–69.2)	0.071
Globally sclerotic glomeruli (%)	15.4 (0.0–29.7)	22.7 (7.7–40.0)	10.0 (0.0–23.1)	**0.002**
Histopathologic classification, n (%)				**<0.001**
Focal	25 (21.9)	4 (7.3)	21 (35.6)	
Crescentic	39 (34.2)	19 (34.5)	20 (33.9)	
Mixed	34 (29.8)	19 (34.5)	15 (25.4)	
Sclerotic	16 (14.0)	13 (23.6)	3 (5.1)	
Arteriosclerosis, n (%)				**0.002**
Grade 0 (0–10%)	45 (39.5)	14 (25.5)	31 (52.5)	
Grade 1 (10–25%)	24 (21.1)	12 (21.8)	12 (20.3)	
Grade 2 (25–50%)	27 (23.7)	14 (25.5)	13 (22.0)	
Grade 3 (>50%)	18 (15.8)	15 (27.3)	3 (5.1)	
TLO formation, n (%)	41 (36.0)	29 (52.7)	12 (20.3)	**<0.001**
Severe tubular atrophy (>50%), n (%)	32 (28.1)	24 (43.6)	8 (13.6)	**<0.001**
Immune complex-mediated glomerulonephritis, n (%)	9 (7.9)	2 (3.6)	7 (11.9)	0.104
Immunofluorescence^a^ >2+, n (%)	19 (26.3)	10 (18.2)	9 (15.3)	0.675

Values are shown as mean ± standard deviation or median (interquartile range).

^a^Moderate to strong positive with antibody to at least one of the following: IgG, IgA, IgM, C3, and fibrinogen.

Abbreviations: ESRD, end-stage renal disease; GFR, glomerular filtration rate; CRP, C-reactive protein; ANCA, anti-neutrophil cytoplasmic antibody; MPO, myeloperoxidase; PR3, proteinase 3; GBM, glomerular basement membrane; TLO, tertiary lymphoid organ.

The median number of glomeruli in the specimens was 16.0 (IQR, 12.0–22.3), and patients with ESRD had a lower proportion of normal glomeruli and a higher proportion of globally sclerotic glomeruli compared with non-ESRD patients (both *P* < 0.05). According to the histopathologic classification, 25 specimens (21.9%) were classified as focal, 39 (34.2%) were crescentic, 34 (29.8%) were mixed, and 16 (14.0%) were sclerotic. Sclerotic specimens were more common among patients who progressed to ESRD, and the focal specimens were more common among patients who did not progress to ESRD. Severe arteriosclerosis, TLO formation, and severe tubular atrophy were more common among patients with ESRD (all *P* < 0.05). Nineteen patients showed >2+ staining with immunofluorescence antibody to at least one of the followings: IgG, IgA, IgM, C3, and fibrinogen. A detailed information on immunofluorescence are shown in S1 Table.

In the logistic regression analysis identifying the relevant factors for TLO formation, moderate to severe tubulitis and moderate to severe interstitial inflammation were associated with TLO formation (both *P* < 0.05) (S2 Table).

### Predictors of renal survival and renal function

**Fig 2:**
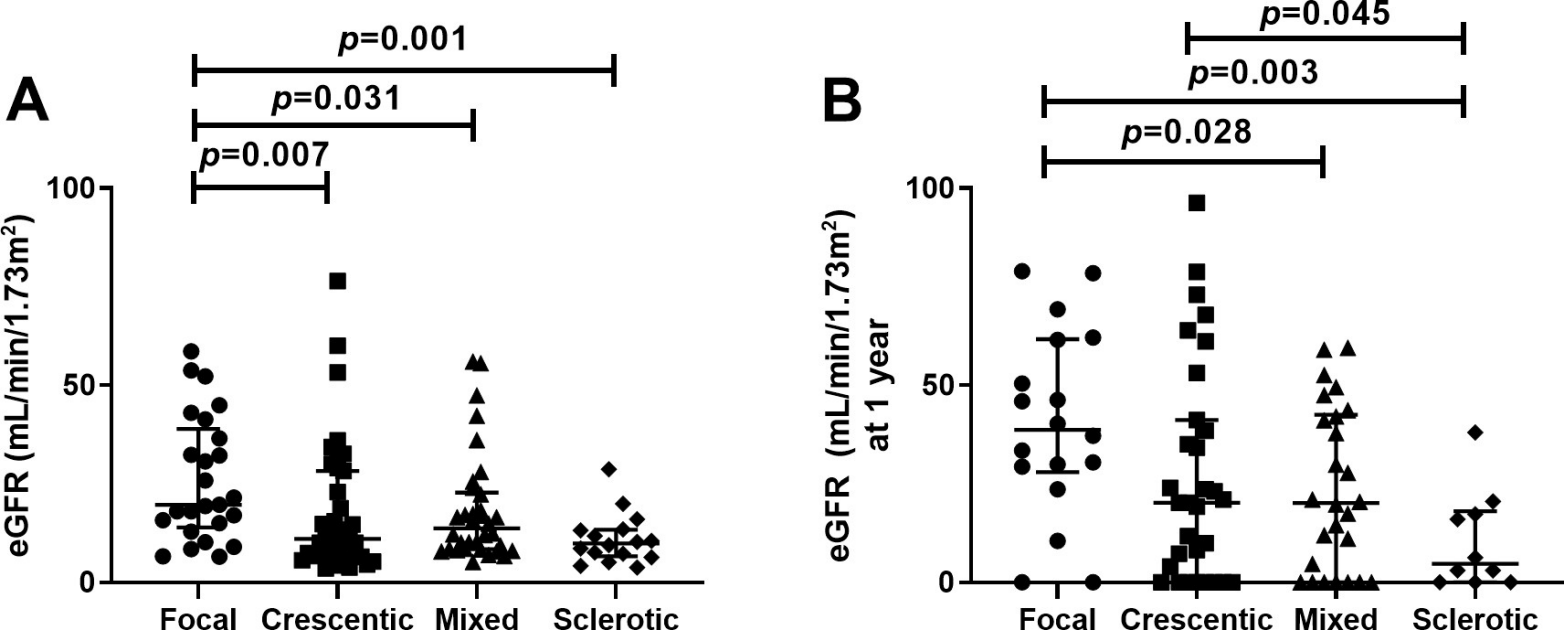
Association between histopathologic classification and estimated GFR. (A) eGFR at diagnosis. (B) eGFR at 1 year after diagnosis. Bars indicate median with interquartile range. Abbreviations: eGFR, estimated glomerular filtration rate.

Fig 2 shows the eGFRs according to the histopathologic classification at the time of diagnosis and 1 year after diagnosis. The mean eGFR at the time of biopsy collection was significantly higher among patients assigned to the focal class compared with the other three classes (all *P* < 0.05), but no differences were observed among the crescentic, mixed, and sclerotic classes (Fig 2A). The mean eGFR 1 year after diagnosis was significantly higher among patients in the focal histologic group compared with those in the mixed (*P* = 0.028) and sclerotic (*P* = 0.003) groups. However, the focal group was not significantly different from the crescentic group 1 year after diagnosis. Additionally, patients in the sclerotic group had a lower mean eGFR at 1 year than patients in the crescentic group (*P* = 0.045) (Fig 2B). Sclerotic histopathology and age were identified as independent predictors of the change in eGFR during 1 year after diagnosis (Table 2).

**Table 2:** Multivariable linear regression analysis of the change in eGFR during 1 year after diagnosis.

Variables	β	SE	*P* value
Age	–0.44	0.11	**<0.001**
Hypertension	–0.46	3.38	0.892
eGFR at biopsy	–0.07	0.12	0.548
Histopathologic classification			
Focal	Reference		
Crescentic	–6.41	4.61	0.167
Mixed	–9.06	4.65	0.054
Sclerotic	–13.48	5.77	**0.022**
Arteriosclerosis			
Grade 0	Reference		
Grade 1	2.01	4.47	0.655
Grade 2	–4.21	4.41	0.343
Grade 3	–1.10	4.90	0.823
TLO formation	5.25	3.31	0.116
Severe tubular atrophy	–5.98	3.76	0.115

Abbreviations: eGFR, estimated glomerular filtration rate; TLO, tertiary lymphoid organ.

**Fig 3:**
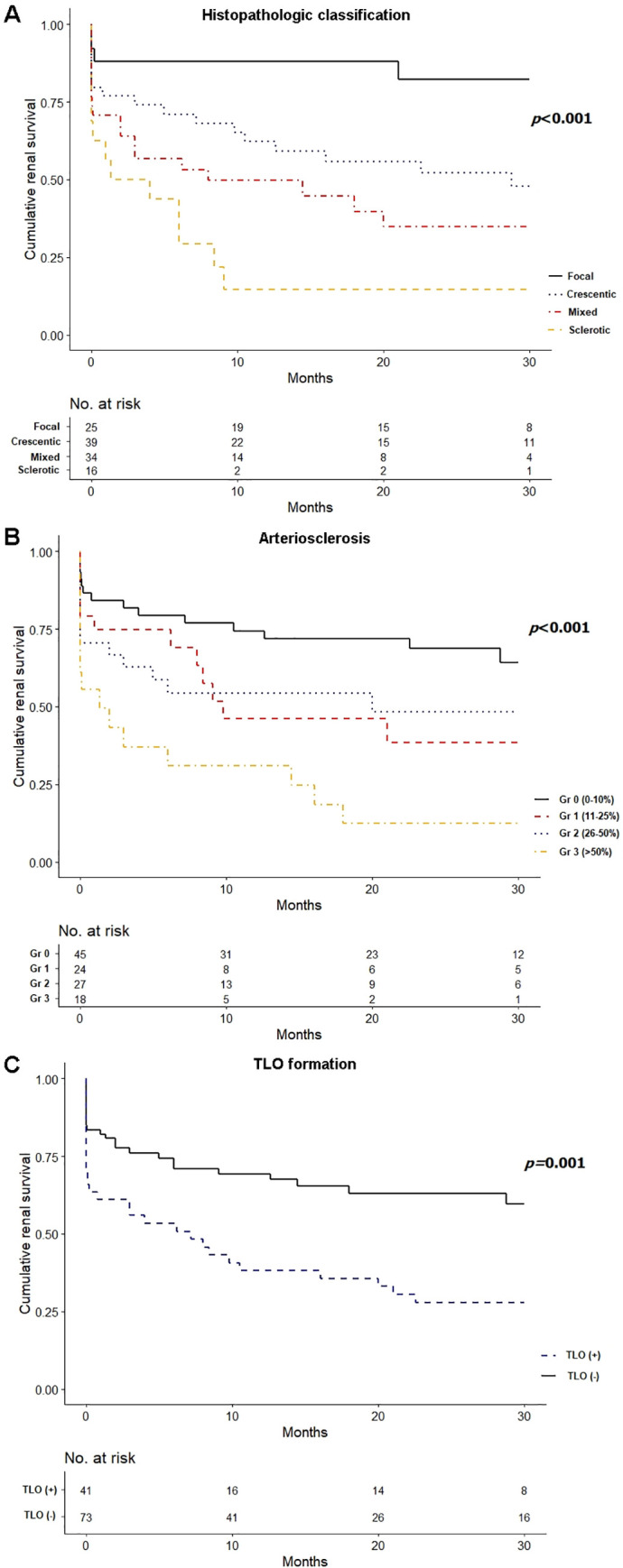
Comparison of renal survival. (A) Renal survival according to histopathologic classification. (B) Renal survival according to severity of arteriosclerosis. (C) Renal survival according to TLO formation. Abbreviations: TLO, tertiary lymphoid organ.

Fig 3 shows the Kaplan–Meier survival curves representing 30-month renal survival according to histopathologic classification, grade of arteriosclerosis, and the presence of TLO formation. A focal histologic categorization was associated with better renal survival compared with the other three classes (log-rank *P* < 0.001), and the sclerotic pattern was associated with poorer renal survival than all the other three classes (all *P* < 0.05) (Fig 3A). Mild to severe arteriosclerosis was associated with poorer renal survival than minimal arteriosclerosis (log-rank *P* < 0.001) (Fig 3B). TLO formation was also a negative predictor of renal survival (log-rank *P =* 0.001) (Fig 3C). In the multivariate Cox regression analysis of renal outcomes, mixed and sclerotic glomerulonephritis (vs. focal: hazard ratio [HR], 3.74; 95% confidence interval [CI], 1.18 to 11.82; *P* = 0.025; and HR, 4.84; 95% CI, 1.44 to 16.32; *P* = 0.011, respectively), severe arteriosclerosis (HR, 2.44; 95% CI, 1.04 to 5.77; *P* = 0.042), TLO formation (HR, 1.82; 95% CI, 1.03 to 3.21; *P* = 0.040), age (HR, 1.03; 95% CI, 1.00 to 1.05; *P* = 0.033), and eGFR at biopsy (HR, 0.92; 95% CI, 0.88 to 0.97; *P* = 0.001) were identified as independent predictors of renal survival (Table 3).

**Table 3:** Predictors for renal survival in Cox proportional hazards model.

Variables	Univariate		Multivariate	
	HR (95% CI)	*P* value	HR (95% CI)	*P* value
Age	1.03 (1.01–1.06)	**0.008**	1.03 (1.00–1.05)	**0.033**
Sex (ref: female)	1.30 (0.76–2.21)	0.336		
Diabetes	0.98 (0.42–2.29)	0.959		
Hypertension	1.67 (0.98–2.84)	0.058	0.86 (0.47–1.57)	0.621
Estimated GFR at biopsy	0.91 (0.87–0.95)	**<0.001**	0.92 (0.88–0.97)	**0.001**
Histopathologic classification				
Focal	Reference		Reference	
Crescentic	3.56 (1.21–10.48)	**0.021**	2.72 (0.87–8.62)	0.084
Mixed	5.05 (1.71–14.90)	**0.003**	3.74 (1.18–11.82)	**0.025**
Sclerotic	9.02 (2.90–28.02)	**<0.001**	4.84 (1.44–16.32)	**0.011**
Arteriosclerosis				
Grade 0	Reference		Reference	
Grade 1	2.08 (0.96–4.52)	0.063	1.37 (0.57–3.25)	0.483
Grade 2	2.08 (0.99–4.37)	0.053	1.24 (0.57–2.73)	0.589
Grade 3	4.40 (2.10–9.22)	**<0.001**	2.44 (1.04–5.77)	**0.042**
TLO formation	2.21 (1.30–3.76)	**0.003**	1.82 (1.03–3.21)	**0.040**
Severe tubular atrophy	2.70 (1.57–4.64)	**<0.001**	0.99 (0.52–1.89)	0.971
Glomerular Ig deposition by IF	1.22 (0.62–2.43)	0.566		

Abbreviations: HR, hazard ratio; CI, confidence interval; GFR, glomerular filtration rate; TLO, tertiary lymphoid organ.; Ig, immunoglobulin; IF, immunofluorescence.

### Predictors of patient survival

**Fig 4:**
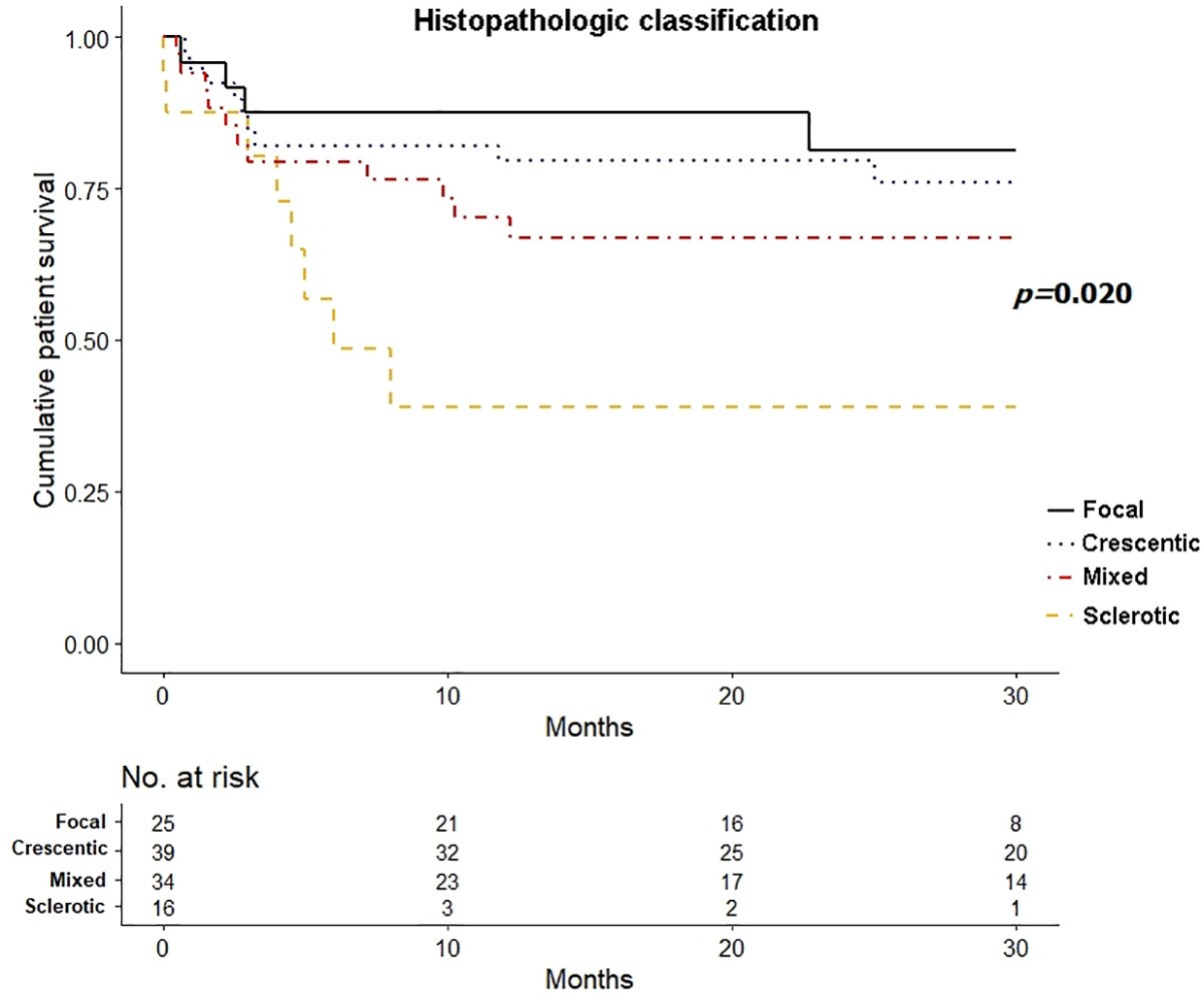
Comparison of patient survival by histopathologic classification.

Thirty-five patients (30.7%) died during the follow-up period, and the death rates were 16.0%, 30.8%, 32.4%, and 50.0% among patients assigned to the focal, crescentic, mixed, and sclerotic groups, respectively. Kaplan–Meier survival analysis for a 30-month mortality revealed that patient in the sclerotic group had the worst survival outlook (log-rank *P* = 0.020) (Fig 4). Additionally, in the multivariate Cox regression analysis, sclerotic glomerulonephritis (vs. focal: HR, 4.96; 95% CI, 1.38 to 17.85; *P* = 0.014) and age (HR, 1.08; 95% CI, 1.04 to 1.12; *P <* 0.001) were independent predictors of death (Table 4).

**Table 4:** Predictors for patient death in Cox proportional hazards model.

Variables	Univariate		Multivariate	
	HR (95% CI)	*P* value	HR (95% CI)	*P* value
Age	1.07 (1.03–1.11)	**0.001**	1.08 (1.04–1.12)	**<0.001**
Sex (ref: female)	0.86 (0.43–1.72)	0.855		
Diabetes	1.05 (0.37–3.00)	0.927	1.17 (0.39–3.51)	0.775
Hypertension	0.61 (0.28–1.33)	0.214	0.68 (0.12–1.54)	0.314
Estimated GFR at biopsy	0.99 (0.96–1.02)	0.451		
ANCA titer	1.26 (0.93–1.71)	0.133		
ANCA type (ref: PR3)	2.20 (0.30–16.27)	0.440		
Histopathologic classification				
Focal	Reference		Reference	
Crescentic	1.45 (0.45–4.70)	0.539	1.87 (0.57–6.11)	0.302
Mixed	2.10 (0.67–6.58)	0.206	2.09 (0.65–6.66)	0.214
Sclerotic	2.89 (0.87–9.62)	0.083	4.96 (1.38–17.85)	**0.014**
Arteriosclerosis				
Grade 0	Reference			
Grade 1	1.46 (0.59–3.57)	0.412		
Grade 2	0.96 (0.38–2.44)	0.931		
Grade 3	1.04 (0.37–2.96)	0.939		
TLO formation	0.74 (0.35–1.57)	0.434		
Interstitial inflammation	0.90 (0.63–1.30)	0.577	0.70 (0.46–1.06)	0.091
Necrotizing lesions in glomeruli	0.55 (0.21–1.43)	0.221	0.81 (0.29–2.23)	0.682

Abbreviations: HR, hazard ratio; CI, confidence interval; GFR, glomerular filtration rate; ANCA, anti-neutrophil cytoplasmic antibody; PR3, proteinase 3; TLO, tertiary lymphoid organ.

## Discussion

This study demonstrated that the histopathologic classification of AAGN, arteriosclerosis, and TLO formation were independent predictors for renal survival among CrGN patients. Results confirmed that the histopathologic classification is a valid predictor for renal outcomes not only among patients with AAGN but also among all patients with CrGN. Our findings are consistent with those of Berden et al. and demonstrate the importance of histopathologic AAGN classification. Other studies have also confirmed that focal glomerulonephritis associated with the best outcomes and the sclerotic variant with the worst renal outcomes among the different histopathologic classes [9–11]. Additionally, this study has suggested that novel histopathologic factors, such as the severity of arteriosclerosis (which reflects the chronic background state of blood flow in the renal parenchyma) and TLO formation (which indicates chronic inflammation status), are important prognostic determinants among CrGN patients.

Similar to other AAGN studies [9, 11], patients assigned to the focal class in this study had significantly better renal survival than patients with mixed and sclerotic glomerulonephritis. The focal pattern was also associated with a non-significant tendency for better renal survival than the crescentic pattern. Additionally, among CrGN patients, the histopathologic category was closely associated with baseline renal function and renal function 1 year after diagnosis [9, 11]. Patients with sclerotic histology not only had worse renal function but also higher mortality than patients with focal glomerulonephritis. The higher mortality rate might be associated with immunosuppression and the higher rate of progression to ESRD among patients with sclerotic glomerulonephritis. As is well known, decreased renal function is an independent risk factor for death [33], and despite treatment, many patients in the sclerotic group progressed to ESRD. These results suggest that clinicians must be cautious with prescribing aggressive immunosuppression for such patients. This study demonstrated that glomerular histopathology is an important predictor of renal function, renal survival, and even patient survival not only among patients with AAGN but also among all CrGN patients, and based on the results, the histopathologic classification used for AAGN should be considered in determining treatment plans for CrGN patients.

Previously, Berden et al. reported that tubulointerstitial parameters did not provide additional benefits in predicting renal outcomes above that provided by the histopathologic classification system for AAGN, and they asserted that tubulointerstitial parameters are unnecessary in the classification system [9]. In contrast, another study from the same group confirmed the importance of tubular lesions for renal outcomes in AAGN [34]. In our study, tubulointerstitial findings provided additional information for renal outcomes in CrGN. CrGN with severe arteriosclerosis, independent of the histologic classification, was associated with a higher risk of progression to ESRD. Arteriosclerosis is associated with hypertension, old age, diabetes, and obesity, and it reflects a state of chronic hypoxia of the renal parenchyma leading to perivascular fibrosis [20, 31, 35–37]. It has also been established that a background of chronic renal arteriosclerosis is indicative of a lower renal reserve to tolerate injury to nephrons caused by CrGN [31, 38]. Although an association between renal arteriosclerosis and the change of eGFR was not found in the early period after diagnosis, it seems to be related that arteriosclerosis itself does not cause rapid renal impairment. In live kidney donor studies, renal arteriosclerosis has been associated with old age, proportion of globally sclerotic glomeruli, and the degree of estimated interstitial fibrosis, but no significant correlation has been found between baseline arteriosclerosis and graft function after short periods of 1 and 3 years after transplantation [39, 40]. Few studies have reported the importance of renal arteriosclerosis in association with AAGN, and none at all in association with CrGN [31, 38]. Additionally, no studies have confirmed the predictive value of arteriosclerosis for renal outcomes alongside the histopathologic classification reflecting glomerular pathology. The severity of renal arteriosclerosis itself at the time of diagnosis, regardless of glomerular pathology, is expected to provide important information about future renal survival among CrGN patients.

There has been a conflicting result on the outcome of AAGN with glomerular immunoglobulin deposition. In this study, no association has been found between immunoglobulin deposition and renal outcome which is consistent to the previous report [41]. However, Haas et al. and Neumann et al. have reported that immunoglobulin deposition is associated with worse renal outcome [42, 43].

Lymphoid neogenesis is caused by chemokines in non-lymphoid organs and reflects chronic inflammation status [17]. In the context of kidney transplantation, TLOs generate effector and memory T cells and provide a fast track for autoimmunity, which perpetuate the rejection process and cause chronic rejection [18, 44]. TLOs also play a detrimental role in IgA nephropathy because they contain all of the essential elements causing local immune responses which thereby activate the immune response [16, 44–46]. Interestingly, one-third of our CrGN patients presented with TLO formation, showing a positive association with active inflammatory tubulointerstitial lesions, such as tubulitis and interstitial inflammation. Thus, TLOs seemed to reflect a chronic active inflammatory status among CrGN patients. Data show that the patients with TLO formation had poor renal survival even after adjusting for other risk factors; this is probably because, TLOs, like other autoimmune diseases, are formed by tissue antigens released by the destructive inflammatory process associated with CrGN [18]. Thus, TLO formation promotes autoimmune response, which is harmful on the renal parenchyma of CrGN patients. In particular, patients with TLO formation progressed to ESRD in the early follow-up period, meaning that these patients needed more aggressive treatment at the time of diagnosis.

CrGN results in high rates of renal failure and patient mortality; thus, clinicians must make prompt and appropriate treatment plans [3]. Treatment plans that balance adequate immunosuppression with the risk of infection prevent the progression of the disease including opportunistic infection. Although only sclerotic glomerulonephritis was considered a predictor of progression to ESRD in the previous CrGN study [4], this study demonstrated that mixed glomerulonephritis (in addition to the sclerotic variant) presents an intermediate risk for renal failure, as shown by other AAGN studies [9, 11]. The histopathologic classification only reflects glomerular histology, but our results emphasized the importance of tubulointerstitial changes in predicting renal outcomes. For the first time, tubulointerstitial information, severity of arteriosclerosis, and TLO formation have been identified as independent predictors of renal survival among CrGN patients. Moreover, among these clinical parameters, age and renal function at the time of diagnosis are well known predictors of future kidney function [10], and they also have been identified as independent predictors for renal outcomes in our study; hence, they should be considered before treatment as well. Interestingly, in the recently published AAGN study, Brix et al. have reported the key predictors for ESRD are as follows: percentage of normal glomeruli, degree of interstitial fibrosis and tubular atrophy, and eGFR at biopsy [47]. Based on our findings, these different results by Brix et al. may be related to racial difference or histopathologic composition. Asians have positivity for MPO-ANCA more frequently than other races [48], and they have worse histopathologic classifications, such as mixed and sclerotic; patients in the sclerotic class in that study were only 5 (5.4%) and those in our study were 16 (14.0%). Hence, personalized treatment plans for CrGN based on individual histopathologic and clinical prognostic factors are recommended to improve renal survival as well as patient survival.

The strength of our study is that this is the first study to confirm that histopathologic classification, which reflects glomerular pathology, can predict renal outcomes among Asian CrGN patients. Tubulointerstitial histopathologic features arteriosclerosis, and TLO formation have been confirmed as novel prognostic predictors. There were some limitations in this study, however. First, this was a multicenter study, so the results may have been affected by the differing CrGN treatment protocols between the centers. However, the protocols were not so different because, in South Korea, the national health insurance system covers most medical care costs, so clinicians treat patients in accordance with the same national health insurance guidelines. Second, because this was a retrospective observational study, it is limited to obtain the effect of individual treatment on patients’ outcomes. This needs to be confirmed by large-scale prospective follow-up studies. Third, although specimens which contain at least 10 glomeruli have been included in this study, sample bias can still be present, particularly when it comes to TLO formation. Fourth, elderly patients might be undertreated due to their general condition or existing comorbid diseases, which may affect the relationship between histopathology and outcome.

In conclusion, this study demonstrated that the specific histopathologic findings, such as histopathologic classification, severity of arteriosclerosis, and TLO formation, provide helpful information in predicting renal outcomes among CrGN patients. Clinical parameters, such as age and renal function at the time of diagnosis, were also identified as independent predictors of renal outcomes. Hence, when patients are diagnosed with CrGN, it is recommended that clinicians establish an individualized treatment plans considering these various predictors.

## Supporting information

S1 Fig Classification of glomerular lesions. (A) Normal glomerulus. (B) Cellular crescent (crescent containing >10% of cellular components). (C) Fibrous crescent (crescent containing ≤10% of cellular components and >90% of extracellular matrix). (D) Globally sclerotic glomerulus (glomerulus containing >80% of sclerotic matrix).
(TIF)

S2 Fig Grading of arteriosclerosis. Grade 0: vascular lumen narrowing of <10%. Grade 1: vascular lumen narrowing of 10% to 25%. Grade 2: vascular lumen narrowing 26% to 50%. Grade 3: vascular lumen narrowing of >50%.
(TIF)

S1 Table Immunofluorescence findings in biopsies. Abbreviation: ESRD, end-stage renal disease.
(DOCX)

S2 Table Associated factors with tertiary lymphoid organ formation. Peritubular capillaritis (ptc): 0, no significant cortical peritubular capillaritis, or <10% of peritubular capillaries with inflammation; 1, ≥10% of cortical peritubular capillaries with capillaritis, with max 3 to 4 luminal inflammatory cells; 2, ≥10% of cortical peritubular capillaries with capillaritis, with max 5 to 10 luminal inflammatory cells; and 3, ≥10% of cortical peritubular capillaries with capillaritis, with max >10 luminal inflammatory cells. Tubulitis (t): 0, no mononuclear cells in tubules; 1, foci with 1–4 cells/tubular cross section (or 10 tubular cells); 2, foci with 5 to 10 cells/tubular cross section; and 3, Foci with .10 cells/tubular cross section. Interstitial inflammation (i): 0, no or trivial interstitial inflammation (<10% of unscarred parenchyma); 1, 10% to 25% of parenchyma inflamed; 2, 26% to 50% of parenchyma inflamed; and 3, >50% of parenchyma inflamed.
(DOCX)

S1 File Data set of the study. (XLSX)
