# Supplementary figures and images for: Novel histopathologic predictors for renal outcomes in crescentic glomerulonephritis
Source: PLoS One. 2020 Jul 27;15(7):e0236051. doi: 10.1371/journal.pone.0236051 (PMC7384637; doi:10.1371/journal.pone.0236051)

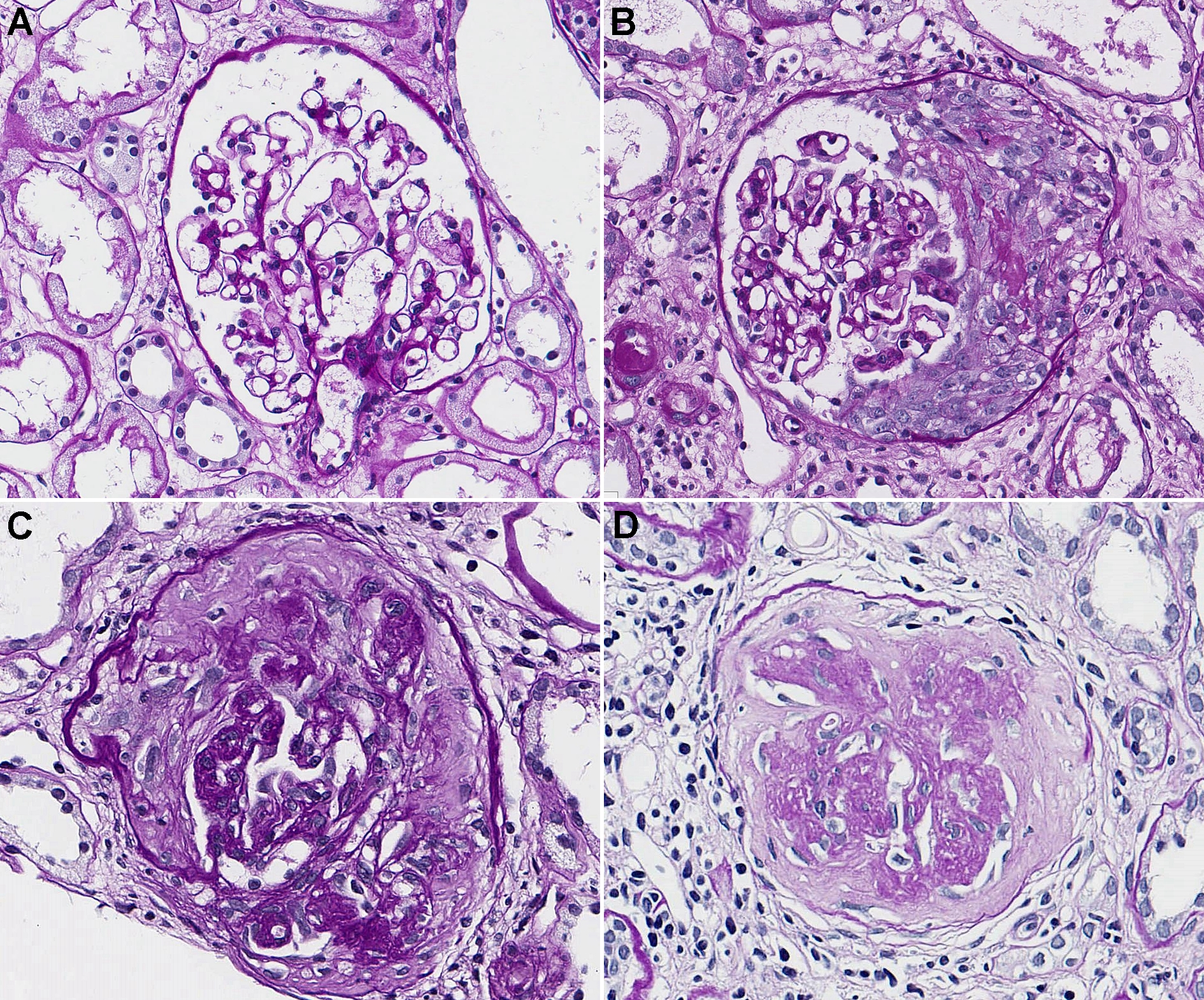

Supplement: S1 Fig — (A) Normal glomerulus. (B) Cellular crescent (crescent containing >10% of cellular components). (C) Fibrous crescent (crescent containing ≤10% of cellular components and >90% of extracellular matrix). (D) Globally sclerotic glomerulus (glomerulus containing >80% of sclerotic matrix). (TIF) [file pone.0236051.s001.tif]

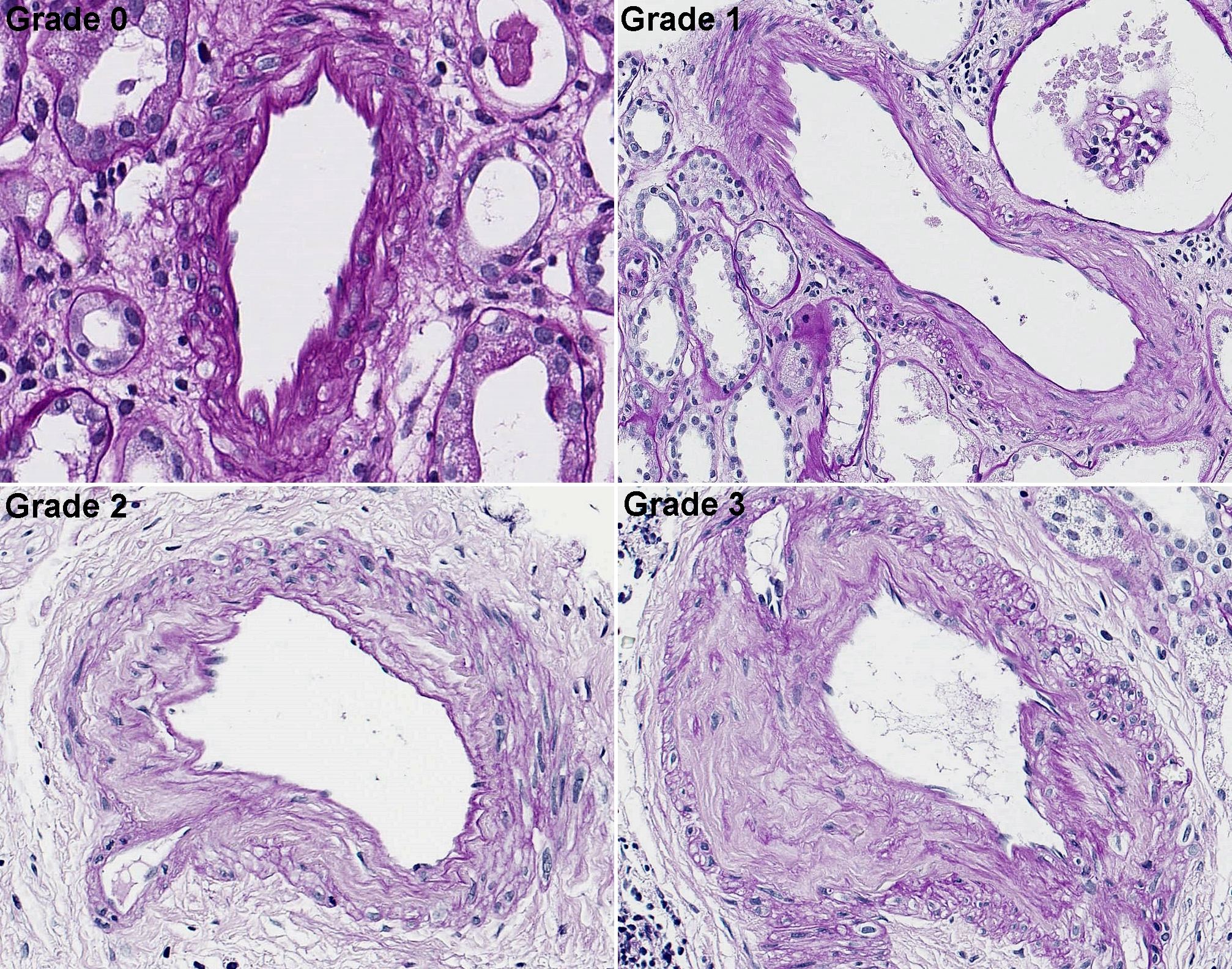

Supplement: S2 Fig — Grade 0: vascular lumen narrowing of <10%. Grade 1: vascular lumen narrowing of 10% to 25%. Grade 2: vascular lumen narrowing 26% to 50%. Grade 3: vascular lumen narrowing of >50%. (TIF) [file pone.0236051.s002.tif]
