# Supplementary material for: Novel histopathologic predictors for renal outcomes in crescentic glomerulonephritis
Source: PLoS One. 2020 Jul 27;15(7):e0236051. doi: 10.1371/journal.pone.0236051 (PMC7384637; doi:10.1371/journal.pone.0236051)
Supplement: S1 Table — Abbreviation: ESRD, end-stage renal disease. (DOCX) [file pone.0236051.s003.docx]

| **Variables** | | **Total (n = 114)** | **ESRD (n = 55)** | **Non-ESRD (n = 59)** | ***P*** |
| --- | --- | --- | --- | --- | --- |
| IgG | ≥1+, n (%) | 30 (26.3) | 15 (27.3) | 15 (25.4) | 0.835 |
|  | ≥2+, n (%) | 14 (12.3) | 7 (12.7) | 7 (11.9) | 0.888 |
|  | >2+, n (%) | 12 (10.5) | 7 (12.7 | 5 (8.5) | 0.460 |
| IgA | ≥1+, n (%) | 22 (19.3) | 9 (16.4) | 13 (22.0) | 0.443 |
|  | ≥2+, n (%) | 12 (10.5) | 6 (10.9) | 6 (10.2) | 0.898 |
|  | >2+, n (%) | 4 (3.5) | 2 (3.6) | 2 (3.4) | 0.943 |
| IgM | ≥1+, n (%) | 18 (15.8) | 9 (16.4) | 9 (15.3) | 0.871 |
|  | ≥2+, n (%) | 6 (5.3) | 4 (7.3) | 2 (3.4) | 0.427 |
|  | >2+, n (%) | 0 | 0 | 0 | NA |
| C3 | ≥1+, n (%) | 49 (43.0) | 23 (41.8) | 26 (44.1) | 0.808 |
|  | ≥2+, n (%) | 21 (18.4) | 8 (14.5) | 13 (22.0) | 0.303 |
|  | >2+, n (%) | 10 (8.8) | 5 (9.1) | 5 (8.5) | 0.907 |
| Fibrinogen | ≥1+, n (%) | 19 (16.7) | 11 (20.0) | 8 (13.6) | 0.357 |
|  | ≥2+, n (%) | 6 (5.3) | 2 (3.6) | 4 (6.8) | 0.680 |
|  | >2+, n (%) | 4 (3.5) | 1 (1.8) | 3 (5.1) | 0.619 |

**S1 Table. Immunofluorescence findings in biopsies**

There were 3 cases of membranoproliferative glomerulonephritis, 5 cases of IgA nephropathy, and 1 case of lupus nephritis.

Abbreviation: ESRD, end-stage renal disease.
