# Supplementary material for: Novel histopathologic predictors for renal outcomes in crescentic glomerulonephritis
Source: PLoS One. 2020 Jul 27;15(7):e0236051. doi: 10.1371/journal.pone.0236051 (PMC7384637; doi:10.1371/journal.pone.0236051)
Supplement: S2 Table — Peritubular capillaritis (ptc): 0, no significant cortical peritubular capillaritis, or <10% of peritubular capillaries with inflammation; 1, ≥10% of cortical peritubular capillaries with capillaritis, with max 3 to 4 luminal inflammatory cells; 2, ≥10% of cortical peritubular capillaries with capillaritis, with max 5 to 10 luminal inflammatory cells; and 3, ≥10% of cortical peritubular capillaries with capillaritis, with max >10 luminal inflammatory cells. Tubulitis (t): 0, no mononuclear cells in tubules; 1, foci with 1–4 cells/tubular cross section (or 10 tubular cells); 2, foci with 5 to 10 cells/tubular cross section; and 3, Foci with .10 cells/tubular cross section. Interstitial inflammation (i): 0, no or trivial interstitial inflammation (<10% of unscarred parenchyma); 1, 10% to 25% of parenchyma inflamed; 2, 26% to 50% of parenchyma inflamed; and 3, >50% of parenchyma inflamed. (DOCX) [file pone.0236051.s004.docx]

| **Variables** | **OR (95% CI)** | ***P* value** |
| --- | --- | --- |
| Age | 1.39 (0.64–2.99) | 0.40 |
| Sex (ref: female) | 1.14 (0.53–2.45) | 0.74 |
| Diabetes | 1.94 (0.63–5.99) | 0.25 |
| Hypertension | 1.94 (0.98–2.84) | 0.10 |
| Moderate to severe peritubular capillaritis (ptc ≥2) | 1.88 (0.65–5.46) | 0.25 |
| Moderate to severe tubulitis (t ≥2) | 3.84 (1.53–9.61) | **0.004** |
| Moderate to severe interstitial inflammation (i ≥2) | 4.29 (1.90–9.70) | **<0.001** |

**S2 Table. Associated factors with tertiary lymphoid organ formation**

Peritubular capillaritis (ptc): 0, no significant cortical peritubular capillaritis, or <10% of peritubular capillaries with inflammation; 1, ≥10% of cortical peritubular capillaries with capillaritis, with max 3 to 4 luminal inﬂammatory cells; 2, ≥10% of cortical peritubular capillaries with capillaritis, with max 5 to 10 luminal inﬂammatory cells; 3, ≥10% of cortical peritubular capillaries with capillaritis, with max >10 luminal inﬂammatory cells. Tubulitis (t): 0, no mononuclear cells in tubules; 1, foci with 1–4 cells/tubular cross-section (or 10 tubular cells); 2, foci with 5 to 10 cells/tubular cross section; 3, Foci with .10 cells/tubular cross section. Interstitial inflammation (i): 0, no or trivial interstitial inflammation (<10% of unscarred parenchyma); 1, 10%–25% of parenchyma inflamed; 2, 26%–50% of parenchyma inflamed; 3, .50% of parenchyma inflamed.

Abbreviations: OR, odds ratio; CI, confidence interval.
